# Deoxycholic Acid at the Interface of Bile Acid Metabolism and Adipose Tissue Remodeling: From Endogenous Signaling to Injectable Adipocytolysis

**DOI:** 10.3390/cimb48100974

**Published:** 2026-09-22

**Authors:** Kyu-Ho Yi, Han Earl Lee, Hong Seok Kim, Thanya Techapichetvanich

**Affiliations:** 1 Division in Anatomy and Developmental Biology, Department of Oral Biology, Human Identification Research Institute, College of Dentistry, Yonsei, 50-1 Yonsei-ro, Seodaemun-gu, Seoul 03722, Republic of Korea; 2 You and I Clinic, 470 Gangnam-daero, Seoul 04524, Republic of Korea; 3 Re & Re Plastic Surgery Clinic, 423 Gangnam-daero, Seoul 04524, Republic of Korea; 4 VOS Dermatology Clinic, 806 Eonju-ro, Seoul 06214, Republic of Korea; 5 Department of Dermatology, Faculty of Medicine Siriraj Hospital, Mahidol University, Bangkok 10700, Thailand

**Keywords:** deoxycholic acid, bile acids, gut microbiome, FXR, TGR5, adipocytolysis, adipose tissue, immunometabolism, lipidomics, metabolomics, ATX-101, submental fat

## Abstract

Deoxycholic acid (DCA) occupies an unusual position at the boundary between endogenous metabolism and therapeutic tissue ablation. Under physiological conditions, DCA is a gut microbial secondary bile acid that enters the enterohepatic circulation, contributes to the composition of the bile-acid pool, and participates in receptor-dependent signaling. When delivered as a concentrated injectable drug into subcutaneous fat, however, DCA acts primarily as an amphipathic cytolytic molecule that disrupts cell membranes, initiates adipocyte death, and provokes an organized inflammatory and reparative response. These two biological identities are often conflated, leading to imprecise descriptions of injectable DCA as a lipolytic, receptor-targeted, or metabolically selective treatment. This critical review integrates evidence from bile-acid biochemistry, microbiology, membrane biophysics, adipose-tissue biology, human histology, pharmacokinetics, and clinical trials. We describe the microbial formation, transport, conjugation, and signaling actions of endogenous DCA; distinguish physiological receptor signaling from concentration-dependent detergent injury; and examine the temporal sequence of adipocytolysis, innate immune recruitment, lipid clearance, fibrovascular remodeling, and volume reduction after local injection. We also assess systemic exposure, clinical efficacy, anatomic determinants of safety, and the limitations of extrapolating submental data to large-volume or non-submental treatment. Finally, we propose a translational metabolomics and lipidomics framework that combines local interstitial sampling, plasma bile-acid profiling, lipid mediator analysis, imaging, and tissue phenotyping. The central conclusion is that injectable DCA is best understood as a controlled local cytolytic perturbation superimposed on a tightly regulated endogenous bile-acid system. Framing the intervention in this way clarifies terminology, identifies measurable biomarkers, and creates a rational path toward dose optimization, patient selection, and safer clinical translation.

## 1. Introduction

Bile acids are frequently introduced as detergents required for intestinal lipid absorption, but this description captures only one part of their biology. They are cholesterol-derived metabolites whose concentrations, conjugation states, microbial transformations, compartmentalization, and receptor affinities collectively influence hepatic metabolism, intestinal physiology, immune responses, and energy balance. Deoxycholic acid (DCA; 3α,12α-dihydroxy-5β-cholan-24-oic acid) is especially instructive because it is simultaneously an endogenous secondary bile acid and the active ingredient of an injectable drug used to reduce submental adipose tissue. The same amphipathic molecular architecture that permits DCA to participate in mixed-micelle formation within the intestinal lumen also enables concentrated DCA to destabilize cellular lipid bilayers when deposited directly into tissue. Understanding DCA therefore requires attention not only to molecular identity, but also to concentration, binding environment, formulation, anatomical compartment, and duration of exposure [1,2,3].

The endogenous and injectable contexts differ by orders of biological organization. Endogenous DCA is generated largely through microbial transformation of cholic acid, absorbed from the intestine, transported to the liver, reconjugated, and returned to bile. Its effects emerge within a dynamic bile-acid pool and are moderated by enterohepatic cycling, protein binding, cellular transporters, and receptor systems. Injectable DCA is formulated at a pharmaceutical concentration and placed into a restricted extracellular volume within subcutaneous fat. At that interface, local membrane exposure can exceed the buffering capacity of surrounding proteins and tissue structures, producing rapid cell-membrane injury rather than classical receptor-mediated metabolic adaptation. The therapeutic response is consequently not simple triglyceride hydrolysis. It is a tissue-level sequence consisting of adipocytolysis, release of intracellular lipid, inflammatory cell recruitment, phagocytic clearance, vascular and stromal repair, and reduction in adipose volume [4,5,6].

Terminology has not always reflected this distinction. The phrases “injection lipolysis,” “fat-dissolving injection,” and “adipocytolysis” are often used interchangeably, even though they refer to different mechanisms. Lipolysis is an enzymatically regulated process in viable adipocytes, involving hydrolysis of stored triacylglycerol into glycerol and non-esterified fatty acids. Cytolysis denotes structural destruction of a cell, with loss of membrane integrity and release of cellular contents. Because concentrated DCA directly injures membranes, adipocytolysis is the more mechanistically accurate term for the primary event. Lipid mobilization and metabolism occur subsequently, during handling of released lipid by macrophages, lymphatic pathways, circulation, and the liver. Maintaining this distinction is essential when interpreting efficacy, systemic safety, and biomarker studies [7,8,9].

Interest in injectable DCA has grown for three related reasons. First, randomized phase III studies established its efficacy for moderate-to-severe submental fullness. Second, clinical practice has expanded toward the jawline and several body regions, where anatomy, fat thickness, dose, and systemic exposure may differ substantially from the approved submental setting. Third, metabolomics, lipidomics, spatial profiling, and high-resolution imaging now provide opportunities to investigate the transition from local membrane injury to tissue remodeling. Nevertheless, the relevant literature remains divided among bile-acid biochemistry, microbial metabolism, receptor biology, aesthetic treatment, pharmacokinetics, and tissue histology.

The need for the present review arises from this separation of disciplines. Reviews of endogenous bile-acid biology generally emphasize microbial transformation, enterohepatic circulation, and receptor signaling, whereas clinically oriented discussions of injectable DCA primarily address contour improvement, injection technique, and adverse events. This separation can obscure the fundamental distinction between regulated physiological signaling and concentration-dependent membrane injury. It may also contribute to imprecise descriptions of injectable DCA as a lipolytic, metabolically selective, or receptor-mediated treatment.

The novelty of this review lies in integrating these domains within a single evidence-conscious framework. Specifically, we connect the microbial and hepatic handling of endogenous DCA with the membrane-level pharmacology of concentrated injectable DCA; distinguish adipocytolysis from classical intracellular lipolysis; organize the tissue response from acute injury through inflammatory clearance and fibrovascular remodeling; and propose a DCA-specific framework linking local exposure, systemic bile-acid handling, molecular measurements, imaging, and clinical outcome. Throughout the review, directly observed DCA-specific evidence is distinguished from mechanistic inference based on broader adipose-injury literature.

## 2. Scope and Literature Approach

This manuscript was developed as a structured critical narrative review. PubMed/MEDLINE-indexed literature, relevant publisher records, regulatory documents, and the reference lists of pivotal studies were examined through June 2026. In PubMed/MEDLINE, the principal DCA-focused search combined (“deoxycholic acid” OR deoxycholate OR “sodium deoxycholate” OR “ATX-101”) with terms representing the major evidence domains: (“bile acid metabolism” OR “secondary bile acid” OR “enterohepatic circulation” OR conjugation OR microbiome OR “7alpha-dehydroxylation”), (FXR OR TGR5 OR GPBAR1 OR signaling), (membrane OR detergent OR cytolysis OR adipocytolysis OR “injection lipolysis”), (“adipose tissue” OR inflammation OR macrophage OR fibrosis OR remodeling), (pharmacokinetics OR “submental fat” OR efficacy OR safety OR “adverse events”), and (metabolomics OR lipidomics OR “bile-acid profiling”). Additional records were identified by examining the reference lists of key mechanistic, histological, pharmacokinetic, and clinical publications and relevant regulatory prescribing information.

Eligible sources included primary studies investigating DCA or deoxycholate; human histological and pharmacokinetic studies of injectable DCA; randomized, prospective, and observational clinical studies; regulatory prescribing information; relevant postmarketing or complication reports; and methodological publications supporting the proposed metabolomics and lipidomics framework. General adipose-injury, macrophage, or extracellular-matrix studies were included only when DCA-specific evidence was unavailable and when they addressed a defined step in the proposed tissue-response sequence. Priority was given to primary experimental studies, controlled clinical trials, human histology, official regulatory information, and established analytical guidance.

Sources were excluded when they were unrelated to DCA biology or injectable adipocytolysis, lacked sufficient methodological relevance, presented unsupported promotional claims, or discussed general adipose metabolism without a direct role in interpreting DCA-induced injury, clearance, or repair. Evidence derived from compounded phosphatidylcholine/deoxycholate mixtures was retained only for mechanistic context and was not treated as pharmacologically interchangeable with a standardized DCA-only injectable formulation. Because this was a critical narrative synthesis rather than a systematic review, the search was not used to generate a PRISMA flow diagram, pooled effect estimate, or formal risk-of-bias score.

Mechanistic conclusions were organized according to the directness of the supporting evidence. Direct observations from human tissue after injectable DCA exposure were given the greatest weight for defining the temporal tissue response. Human pharmacokinetic studies and controlled clinical trials informed systemic exposure, efficacy, and clinically documented adverse events. Animal and ex vivo studies provided supporting evidence regarding membrane injury and tissue toxicity when corresponding human mechanistic data were unavailable. Cell-culture studies were used to clarify concentration-dependent cytotoxicity and the absence of intrinsic adipocyte-specific selectivity but were not considered direct evidence of in vivo tissue selectivity. Evidence from obesity-associated adipocyte death, macrophage biology, inflammation, or fibrosis was used only to develop explicitly identified mechanistic hypotheses. This hierarchy was applied throughout the review to avoid presenting biological plausibility as established DCA-specific human evidence.

## 3. DCA as an Endogenous Microbial–Host Metabolite

### 3.1. Formation from Cholesterol-Derived Primary Bile Acids

Primary bile acids are synthesized from cholesterol in hepatocytes through coordinated enzymatic pathways. In humans, cholic acid and chenodeoxycholic acid are the principal primary bile acids. Before biliary secretion, these molecules are commonly conjugated with glycine or taurine, which lowers their apparent pKa, increases their aqueous behavior at intestinal pH, and supports their participation in dietary lipid digestion and absorption. Bile-acid synthesis also provides an important route for cholesterol disposal, while feedback regulation limits excessive accumulation of these potentially membrane-active molecules [1,3].

DCA is 3α,12α-dihydroxy-5β-cholan-24-oic acid. Its rigid steroid nucleus contains hydroxyl groups at the 3α and 12α positions and a carboxyl-containing side chain. The spatial arrangement of these hydrophilic groups on one surface of the steroid nucleus, opposite a comparatively hydrophobic surface, produces the facial amphipathicity that underlies both its physiological detergent behavior and its capacity to disrupt phospholipid membranes at sufficiently high local concentrations [1,2,3].

DCA is formed predominantly after host-derived bile reaches the intestine. Conjugated cholic acid is first deconjugated by microbial bile-salt hydrolase activity. A limited group of anaerobic intestinal bacteria then converts cholic acid to DCA through a multistep 7α-dehydroxylation pathway. Classical studies established the microbial basis of this transformation, while later biochemical work further characterized the pathway and its intermediates [10,11]. DCA is subsequently reabsorbed, transported to the liver through the portal circulation, extracted by hepatocytes, reconjugated principally with glycine or taurine, and resecreted into bile as part of the enterohepatic pool. Thus, DCA abundance reflects both host production of cholic acid and microbial deconjugation and 7α-dehydroxylation, together with intestinal transit, diet, absorption, and hepatic recycling.

The organisms responsible for DCA formation may be low in relative abundance but metabolically influential. A 2024 human study associated production of DCA by low-abundance microbial species with impaired glucose metabolism, emphasizing that microbial function cannot be inferred reliably from taxonomic abundance alone [12]. Such associations do not establish that DCA is uniformly harmful; rather, they demonstrate that systemic metabolic phenotype, microbial context, and total bile-acid composition shape the meaning of any single metabolite measurement. DCA can be a marker of microbial transformation, a signaling ligand, a membrane-active molecule, and an exposure linked to disease risk, depending on compartment and concentration.

### 3.2. Enterohepatic Circulation, Conjugation, and Compartmentalization

Most intestinal DCA is reabsorbed and returned to the liver through the portal circulation. Hepatocytes efficiently extract bile acids, reconjugate free species, and resecrete them into bile. Only a small fraction of the total pool escapes reabsorption and is excreted in feces; that loss is balanced by de novo hepatic synthesis. Repeated cycling creates high luminal and biliary concentrations while maintaining much lower systemic concentrations. This compartmentalization is biologically protective, because bile acids that are useful in the intestinal lumen can be injurious when present at inappropriate concentrations near cellular membranes [2,3].

Conjugation changes physicochemical behavior and receptor pharmacology. Free DCA, glycodeoxycholic acid, and taurodeoxycholic acid are related but not interchangeable analytes. Their relative proportions depend on hepatic conjugation, microbial deconjugation, species-specific metabolism, diet, and disease. For translational studies of injectable DCA, measuring only “total DCA” can obscure whether the circulating signal reflects the administered unconjugated molecule, rapid incorporation into the endogenous pool, or secondary changes in conjugated species. Targeted assays should therefore quantify individual unconjugated and conjugated bile acids and report the biological matrix, collection time, fasting status, and preanalytical handling.

Protein binding also separates physiological circulation from local injection. The approved injectable formulation is deposited into extracellular tissue fluid before full equilibration with plasma proteins. In blood, DCA is extensively protein-bound, and the surrounding protein concentration can attenuate detergent activity. In subcutaneous fat, the immediate microenvironment contains extracellular matrix, interstitial proteins, microvessels, nerves, adipocytes, stromal cells, and variable water content. Local diffusion and binding determine which membranes are exposed to unbuffered drug. Thus, total administered dose alone does not predict tissue effect; concentration, injection volume, spacing, tissue thickness, and protein-rich boundaries are equally important.

### 3.3. FXR Signaling and Feedback Regulation of the Bile-Acid Pool

The farnesoid X receptor (FXR; NR1H4) is a nuclear receptor activated by bile acids. Landmark studies identified bile acids as endogenous FXR ligands and established a receptor-mediated framework for bile-acid feedback control [13,14,15]. FXR activation in the liver and intestine regulates genes involved in bile-acid synthesis, transport, conjugation, and lipid metabolism. In the intestine, FXR-driven fibroblast growth factor 19 in humans—FGF15 in mice—acts as an enterohepatic signal that suppresses hepatic bile-acid synthesis [16,17]. These pathways prevent uncontrolled expansion of the bile-acid pool and coordinate digestive demand with hepatic synthesis.

DCA can participate in FXR-related biology, but it should not be described as the universal or dominant FXR ligand in every context. Bile acids differ in receptor potency, and the effective signal depends on concentration, conjugation, transporter expression, tissue exposure, and competition within the pool. Moreover, microbial conversion of primary to secondary bile acids can alter the balance of agonistic and antagonistic species. In mice, microbiota-dependent changes in tauro-β-muricholic acid regulate intestinal FXR signaling, illustrating how bacterial metabolism can reshape receptor tone without requiring a change in total bile-acid concentration [18]. Human bile-acid composition differs from that of mice, so direct extrapolation requires caution.

For injectable DCA, FXR signaling is unlikely to explain the immediate local therapeutic effect. Nuclear receptor responses require viable cells, ligand transport, transcriptional regulation, and time. Histological injury begins rapidly and is consistent with physical membrane disruption. Receptor signaling may still influence surviving stromal, endothelial, immune, or hepatic cells after exposure, but it should be considered a downstream or systemic modifier rather than the proximate cause of adipocyte destruction. This distinction is central to interpreting molecular studies: detection of an FXR-responsive gene after DCA exposure does not prove that the same pathway mediates local cytolysis.

### 3.4. TGR5 and Metabolic Signaling Beyond the Nucleus

The G-protein-coupled bile-acid receptor TGR5, also known as GPBAR1, provides a second major signaling axis. Independent studies identified a membrane receptor responsive to bile acids and demonstrated expression across multiple tissues [19,20]. TGR5 activation increases intracellular cyclic AMP and can influence energy expenditure, glucose handling, immune-cell behavior, and enteroendocrine signaling. In brown adipose tissue and skeletal muscle, bile-acid-induced activation of type 2 iodothyronine deiodinase has been linked to increased energy expenditure in experimental models [21]. TGR5 signaling in intestinal L cells can promote glucagon-like peptide-1 secretion and improve glucose homeostasis in mice [22].

DCA is a relatively active TGR5 ligand, which has encouraged descriptions of it as an energy-regulating or anti-inflammatory metabolite. Yet receptor activation and cytotoxicity occupy different exposure domains. Nanomolar-to-micromolar receptor pharmacology in a protein-containing physiological environment cannot be equated with the local conditions produced by injection of a 10 mg/mL formulation. At high local concentrations, membrane injury can precede or eliminate regulated signaling. In addition, TGR5 effects are cell-type specific: cyclic-AMP signaling in an enteroendocrine cell, macrophage, cholangiocyte, or adipocyte does not necessarily produce the same physiological outcome.

Experimental studies also reveal that the metabolic consequences of bile-acid signaling and DCA exposure are context-dependent. Pharmacological activation of TGR5 and FXR can improve selected metabolic phenotypes, and direct DCA administration has recently been linked to enhanced thermogenesis and improved glucose homeostasis in mice; conversely, increased DCA-generating microbial activity has been associated with insulin resistance, inflammatory signaling, and carcinogenic pathways [23,24,25]. These apparently conflicting observations are not necessarily contradictory. Dose, route, duration, microbial ecology, diet, host genotype, and target organ differ. The key translational lesson is that “DCA exposure” is not a single biological state.

### 3.5. DCA as a Context-Dependent Metabolic Signal and Stressor

Secondary bile acids can function as beneficial signals at one concentration and cellular stressors at another. DCA has been reported to influence mitochondrial function, oxidative stress, endoplasmic reticulum responses, inflammatory pathways, and cell survival. In hepatocytes and other epithelial contexts, prolonged or excessive DCA exposure may promote injury, while receptor-mediated signaling can modulate immunity and metabolism. The net result reflects membrane exposure, intracellular accumulation, detoxification capacity, and the presence of other bile acids.

This duality is relevant to injectable therapy in two ways. First, a local pharmaceutical dose is intentionally used to cross a cytotoxic threshold within a defined tissue volume. Second, the drug subsequently enters a systemic pool that is already accustomed to handling endogenous DCA. Pharmacokinetic safety therefore depends on whether local administration produces a transient increment that remains within the buffering and transport capacity of the enterohepatic system. Existing studies support rapid return toward endogenous concentrations after approved submental dosing, but they do not establish safety for unlimited dose escalation or large-area body treatment.

Table 1 summarizes the major differences between endogenous DCA and injectable DCA. The two contexts share a molecule but not an exposure geometry. Endogenous DCA is distributed through regulated compartments and interpreted through receptors, transporters, conjugation, and microbial–host cycling. Injectable DCA begins as a high-concentration local depot designed to damage membranes. Conflating them obscures both the physiology and the therapy.

## 4. From Signaling Metabolite to Injectable Cytolytic Agent

### 4.1. Amphipathic Structure and Membrane Solubilization

DCA contains a rigid steroid nucleus with hydrophilic hydroxyl and carboxyl groups positioned on one face and a comparatively hydrophobic surface on the other. This facial amphipathicity differs from the flexible head-and-tail architecture of many conventional surfactants, but it still enables interaction with phospholipid membranes and formation of mixed aggregates. At sufficient concentration, bile salts partition into lipid bilayers, alter packing, increase permeability, and ultimately solubilize membrane components into mixed micelles. Detergent-mediated bilayer solubilization is not an instantaneous binary event; it can proceed through membrane saturation, destabilization, fragmentation, and mixed-micelle formation, with thresholds influenced by lipid composition, temperature, ionic conditions, and protein content [26].

Early investigation of phosphatidylcholine/deoxycholate formulations demonstrated that sodium deoxycholate was a major driver of cell destruction. Rotunda and colleagues showed prominent detergent effects and helped shift the field from the concept of a phosphatidylcholine-specific metabolic action toward a deoxycholate-mediated cytolytic mechanism [4]. Subsequent animal, ex vivo, and cell-culture studies demonstrated membrane injury, inflammation, and tissue toxicity after phosphatidylcholine/deoxycholate exposure [27,28]. These studies remain important historically, although compounded phosphatidylcholine/deoxycholate mixtures should not be assumed to have identical pharmacology to a standardized DCA-only injectable product.

In vitro, deoxycholate can injure multiple cell types rather than selectively recognizing adipocytes. Janke and colleagues reported destruction of adipocytes and other cells found in adipose tissue, and Klein et al. concluded that the observed effect was better characterized as lipid dissolution/cell destruction than regulated lipolysis [8,9]. Thuangtong et al. provided a mechanistic explanation for apparent tissue selectivity: protein-rich tissues and albumin can attenuate deoxycholate cytotoxicity, while adipose tissue may provide a microenvironment in which adipocytes are preferentially exposed [5]. Selectivity is therefore relative and anatomical, not the result of an adipocyte-specific receptor.

### 4.2. Concentration, Formulation, and the Local Exposure Field

The approved DCA formulation contains 10 mg/mL DCA and is administered as 0.2 mL aliquots spaced approximately 1 cm apart in preplatysmal submental fat. At the labeled area-adjusted dose of 2 mg/cm^2^, each injection creates a local concentration gradient rather than a uniform bath [29]. The central region of a depot may experience rapid membrane-disrupting exposure, while peripheral tissue receives lower concentrations through diffusion and dilution. Adjacent depots can overlap, making injection spacing a determinant of cumulative injury.

Four related parameters should be distinguished: concentration, volume per injection, dose per unit area, and total dose. Concentration influences the probability that a membrane-solubilizing threshold will be reached. Volume determines the initial size and geometry of the depot. Dose per area influences overlap among depots and the completeness of treatment across the target. Total dose determines the amount potentially available for systemic absorption. Two protocols with the same total dose can therefore produce different local effects if one uses fewer high-volume depots and the other uses more evenly spaced microdeposits.

Formulation also matters. DCA, sodium deoxycholate, and phosphatidylcholine/deoxycholate mixtures are frequently grouped together in reviews, but they differ in excipients, pH, micellar behavior, regulatory quality, and relative concentrations. The approved product should not be diluted or mixed with other compounds according to prescribing information [29]. Data derived from compounded mixtures are valuable for understanding membrane injury, but their adverse-event profiles cannot be transferred uncritically to a standardized product.

Tissue architecture shapes the exposure field. Superficial injection can expose the dermis and create ulceration or necrosis; deep injection can expose platysma, glandular tissue, vessels, or nerves. Fibrous septa may constrain or redirect diffusion. Scar tissue from previous procedures can distort planes and alter mechanical resistance. Vascular puncture may change distribution and increase bruising or tissue injury. These are not merely technical complications added onto a pharmacological mechanism; they are determinants of where the pharmacological mechanism occurs.

### 4.3. Adipocytolysis Is Not Classical Lipolysis

Classical lipolysis occurs within living adipocytes through regulated activation of adipose triglyceride lipase, hormone-sensitive lipase, and monoglyceride lipase. Catecholamines, insulin, natriuretic peptides, and intracellular signaling pathways govern the rate at which stored triacylglycerol is hydrolyzed [7]. The adipocyte membrane remains intact, and released fatty acids and glycerol exit through regulated transport and diffusion.

DCA injection produces a different initiating event. Membrane disruption causes loss of ionic homeostasis, leakage of intracellular components, and cell death. Neutral lipid droplets may persist transiently after the cell has lost viability, creating extracellular lipid pools that require phagocytic and lymphatic clearance. Some enzymatic hydrolysis undoubtedly occurs during this process, but it is secondary to structural injury. Calling the treatment “lipolysis” implies selective metabolic activation of viable adipocytes and may understate risks to non-adipocyte structures.

A more precise sequence is DCA deposition, membrane interaction, adipocyte and local cellular injury, release of intracellular lipid and damage-associated molecular patterns, acute inflammation, macrophage-dominated clearance, stromal and vascular repair, and reduction in fat-lobule volume. Figure 1 integrates the endogenous and injectable contexts of DCA, whereas Figure 2 distinguishes regulated receptor-level signaling from concentration-dependent membrane disruption and cytolysis. These figures are conceptual syntheses; the detailed chemical, cellular, and evidentiary qualifications are provided in Section 3, Section 4 and Section 5 and Tables 1–3.

## 5. Local Adipose-Tissue Response After Injection

### 5.1. Immediate Injury and Early Inflammation

Human histological data provide the most direct description of the tissue response. In a phase I open-label study, subcutaneous adipose samples obtained at defined intervals after ATX-101 injection showed a reproducible temporal pattern. Early samples demonstrated adipocytolysis and acute inflammatory changes, followed by macrophage infiltration and later fibrovascular remodeling [6]. The study was small and involved abdominal tissue rather than the submental compartment, but it remains a critical bridge between biophysical mechanism and clinical outcome.

Within the early phase, membrane injury generates extracellular lipid, cellular debris, ions, nucleotides, and other damage-associated molecular patterns. Neutrophils and monocytes are recruited to the injured site, while local vessels may show congestion, hemorrhage, or reactive change. Injection itself also produces mechanical trauma; therefore, not every early inflammatory feature can be attributed exclusively to DCA. Placebo-controlled histology would be required to quantify the specific contribution of needle injury, vehicle, and active drug.

The clinical correlate is predictable swelling, tenderness, bruising, erythema, and induration. These events are often categorized as adverse reactions, but they also reflect the intended biological process. The therapeutic challenge is to produce sufficient local injury for adipose reduction without allowing the exposure field to include skin, muscle, nerve, gland, or vessel. The boundary between expected inflammation and tissue complication is determined by severity, duration, anatomical structure involved, and functional consequence.

### 5.2. Macrophage Recruitment and Lipid Clearance

Human histology after ATX-101 exposure provides direct evidence that acute adipocytolysis is followed by macrophage infiltration and later fibrovascular change [6]. The available human evidence establishes the presence and timing of this cellular response but does not yet define the complete molecular phenotype of the recruited macrophages or quantify the relative contribution of individual lipid clearance pathways.

Evidence from other adipose-injury settings provides contextual support rather than direct DCA-specific proof. In obesity-associated adipocyte death, macrophages localize around injured adipocytes and acquire inflammatory and lipid-processing characteristics [30,31]. Experimental work also shows that adipocyte death can activate resident macrophages and induce a pro-inflammatory metabolic program [32]. These observations provide a plausible framework for interpreting macrophage infiltration after DCA-induced adipocytolysis, but differences in exposure, time course, anatomical setting, and metabolic background prevent direct equivalence.

After DCA injection, extracellular neutral lipid may undergo local hydrolysis, macrophage uptake, lymphatic drainage, transfer to circulating lipoproteins, and hepatic processing. The relative contribution of these routes has not been quantified in humans. Conventional serum studies did not identify clinically meaningful lipid changes after approved dosing [33], but these measurements may not detect brief changes in non-esterified fatty acids, glycerol, oxylipins, lysophospholipids, acylcarnitines, or lipoprotein composition. Detailed macrophage-state transitions and local lipid-processing pathways should therefore be regarded as testable mechanisms rather than established human DCA findings.

Human histology after ATX-101 has demonstrated later septal thickening, neovascularization, fat-lobule atrophy, and reduced inflammation by approximately four weeks [6]. These findings support an organized subcutaneous repair response. Evidence concerning collagen VI, metabolically unhealthy adipose fibrosis, and adaptive extracellular-matrix remodeling derives primarily from broader adipose-biology studies [34,35,36]. Such evidence may help interpret the consequences of local tissue injury but does not establish the same molecular pathway after injectable DCA.

Similarly, profibrotic adipose progenitor states and PDGFRα-associated cellular transitions have been described in obesity-related fibrosis [37], but their involvement after DCA injection has not been demonstrated. Because deoxycholate can injure non-adipocyte cells in vitro, the survival and behavior of progenitor and stromal populations may vary according to distance from the injection depot, tissue protein content, and local exposure. Spatially resolved studies are required before these mechanisms can be assigned specifically to injectable DCA.

Improvement in contour after fat reduction should not be equated with established dermal neocollagenesis or skin rejuvenation. Current human evidence supports subcutaneous septal thickening and fibrovascular remodeling but does not establish clinically meaningful dermal collagen synthesis after DCA injection. Matrix change may contribute to contour stabilization, whereas excessive or disorganized remodeling could contribute to persistent induration, nodularity, irregularity, or difficulty during subsequent surgery.

### 5.3. Temporal Model of Tissue Remodeling

A practical temporal model can be organized into four overlapping phases. Phase 1, occurring over minutes to approximately one day, includes depot formation, membrane disruption, vascular permeability, and acute inflammatory signaling. Phase 2, over several days, includes neutrophil activity, monocyte recruitment, macrophage lipid uptake, and removal of cellular debris. Phase 3, over one to four weeks, includes resolution of acute inflammation, angiogenesis, fibroblast activity, septal remodeling, and progressive reduction in fat-lobule volume. Phase 4, over subsequent months, includes maturation of the repaired matrix and stabilization of contour (Figure 3).

This model should not be interpreted as a rigid timetable. Dose, region, tissue thickness, treatment history, host metabolic health, and local vascularity can shift each phase. Larger depots may prolong inflammation. Thin fat compartments may expose non-target structures. Previously treated tissue may have altered compliance and diffusion. Patients with diabetes, smoking exposure, impaired wound healing, immunosuppression, or vascular disease were not the dominant populations in pivotal trials, so their tissue-response kinetics may differ.

Table 2 integrates human histological observations with mechanistic interpretation and candidate biomarkers. Some proposed biomarkers are hypothesis-generating rather than validated. Their purpose is to guide future studies, not to imply established clinical utility.

### 5.4. Anatomical and Host Determinants of Response

The submental region contains preplatysmal and postplatysmal fat, platysma, submandibular glands, lymph nodes, vessels, and the marginal mandibular nerve. Clinical benefit depends on treating sufficient preplatysmal fat while avoiding vulnerable structures. The chin and adjacent fat compartments are anatomically heterogeneous, and apparent fullness may arise from skin laxity, platysmal banding, retrognathia, skeletal contour, gland prominence, or deeper fat rather than a readily injectable superficial adipose target [38].

Baseline fat thickness influences both efficacy and safety. Very thin compartments reduce the margin between dermis and muscle. Thick compartments may require more treatment points or sessions, increasing total exposure. Fibrous density influences injectability and diffusion. Sex, age, BMI, insulin resistance, and prior weight change may alter adipocyte size, macrophage burden, fibrosis, and microvascular function. Yet clinical dose selection remains primarily anatomical and visual rather than metabolically individualized.

Host bile-acid physiology may affect systemic exposure but is rarely measured. Fasting state, recent meals, microbiome composition, antibiotic use, bile-acid sequestrants, hepatic disease, and bariatric surgery can alter baseline DCA concentrations. Because endogenous DCA is highly variable, a post-injection concentration without a baseline time series may be difficult to interpret. Future pharmacokinetic studies should use standardized meals or fasting, repeated baseline samples, and measurement of the broader bile-acid pool.

## 6. Pharmacokinetics and Systemic Metabolic Handling

### 6.1. Absorption into an Endogenous Pool

Pharmacokinetic interpretation is complicated by the fact that DCA is present before treatment. Following the maximum recommended single treatment dose of 100 mg, the prescribing information reports rapid systemic absorption, a median time to maximum concentration of approximately 18 min, a mean peak concentration about 3.2-fold above average endogenous peak values, and return to the endogenous range within 24 h [29]. The injected molecule joins the endogenous bile-acid pool and is eliminated principally through normal enterohepatic and fecal pathways rather than extensive xenobiotic metabolism.

A phase I study similarly found a transient increase in plasma DCA and no evidence of accumulation with the proposed treatment interval [39]. A related study did not identify clinically meaningful changes in conventional serum lipids, adipokines, or inflammatory cytokines after treatment [33]. These findings support the metabolic tolerability of labeled submental dosing in studied adults. They do not prove that all local lipid products remain confined to tissue, only that the systemic perturbation was not apparent in the selected measurements and sampling scheme.

Several limitations deserve emphasis. Sample sizes were small, participants were selected, and the number of analytes was modest compared with contemporary metabolomic capabilities. Baseline DCA varies within and between individuals. Postprandial bile-acid excursions can overlap with drug-related changes. Conventional lipid panels are insensitive to many transient intermediates. Furthermore, the approved maximum of 100 mg represents a small fraction of the total bile-acid pool, whereas off-label treatment of broad body areas could involve different total doses, depot numbers, and absorption kinetics.

### 6.2. Local Lipid Release Versus Systemic Lipid Burden

Destruction of adipocytes releases stored triacylglycerol, but the absence of a large serum triglyceride signal is biologically plausible. Neutral lipid is poorly soluble and may remain within disrupted tissue until emulsified, hydrolyzed, phagocytosed, or drained. Macrophages can sequester lipid and process it gradually. Lymphatic transport and hepatic clearance can distribute the load over time. The amount of fat removed from a localized submental area is also small relative to whole-body adipose mass and daily dietary lipid flux.

Nonetheless, systemic safety should not be inferred solely from the visual size of the treated region. The relevant variables are mass of destroyed adipose tissue, rate of lipid release, local inflammatory burden, DCA dose, and the patient’s capacity to handle fatty acids and bile acids. Individuals with severe hypertriglyceridemia, advanced liver disease, cholestasis, impaired lymphatic drainage, or poorly controlled metabolic disease may respond differently, although direct evidence is limited. Future studies should report liver enzymes, apolipoproteins, non-esterified fatty acids, acylcarnitines, ketone bodies, and bile-acid species in addition to conventional lipids.

### 6.3. Potential Interaction with Metabolic Phenotype

Adipose tissue in insulin-resistant obesity is often inflamed and fibrotic, with impaired lipid buffering. A DCA-induced injury in such tissue may produce a different cytokine and lipidomic response than in metabolically healthy adipose tissue. Conversely, thicker adipose tissue may better confine the depot and protect non-target structures. These competing effects have not been resolved. Clinical trials included participants across a range of BMI values but were not designed to stratify outcomes by insulin sensitivity, liver fat, microbiome, or baseline bile-acid profile.

A mechanistically informative study would therefore enroll participants across predefined metabolic phenotypes and use identical area-adjusted dosing. Baseline characterization could include fasting glucose, insulin, hemoglobin A1c, lipids, liver enzymes, body composition, and targeted bile-acid profiling. Such stratification would determine whether metabolic health predicts swelling, clearance kinetics, efficacy, or adverse events. It would also clarify whether DCA injection is metabolically neutral because the treated mass is small or because local and systemic buffering mechanisms are robust across phenotypes.

## 7. Clinical Translation: Efficacy, Technique, and Safety

### 7.1. Evidence for Submental Fat Reduction

European phase III studies demonstrated that ATX-101 reduced clinician- and patient-rated submental fat compared with placebo, with dose-related local reactions [40,41,42]. The North American REFINE-1 and REFINE-2 trials confirmed the efficacy of 2 mg/cm^2^ DCA for moderate or severe submental fullness, including improvements on validated rating scales and magnetic-resonance-based fat-volume assessment [43,44]. A pooled analysis showed progressive response across treatment sessions, supporting individualized cessation once the desired result was achieved [45]. Follow-up data suggest that contour improvement is maintained for several years in many responders, consistent with permanent removal of a population of adipocytes rather than transient dehydration or acute contraction [46].

The magnitude of benefit is clinically meaningful but not equivalent to surgical fat removal. Multiple sessions may be required, and the treatment course includes predictable swelling. Patient satisfaction depends on pretreatment counseling, tolerance for downtime, baseline anatomy, and realistic expectations. DCA is most rational for a localized preplatysmal adipose target in a patient who prefers a non-surgical approach and does not have dominant skin laxity, prominent platysmal bands, glandular fullness, or skeletal deficiency.

Real-world studies have reported outcomes broadly consistent with clinical trials while emphasizing treatment customization and experience-related reduction in adverse events [47,48,49]. These reports are useful for technique and patient selection but are more susceptible to selection, assessment, and publication bias than randomized trials. They should complement, not replace, controlled evidence.

Table 3 separates the controlled evidence for approved submental treatment from longer-term and real-world observations. Quantitative details are limited to information reported in the evidence summarized in this review, including the labeled area-adjusted dose, maximum number of injections, treatment interval, and reported follow-up duration. Off-label regional use is addressed separately in Section 7.4 because its heterogeneous anatomy, dosing, and outcome assessment do not permit direct comparison with the controlled submental evidence.

### 7.2. Injection Geometry as a Biological Variable

The labeled approach uses 0.2 mL injections at 1 cm spacing, with up to 50 injections per session and at least one month between sessions [29]. These parameters create a reproducible exposure field and allow inflammation to resolve before retreatment. The grid is not merely a convenience; it standardizes depot density. Deviating from concentration, injection volume, or spacing changes tissue pharmacodynamics.

Correct plane selection is equally important. The target is preplatysmal subcutaneous fat, approximately midway between dermis and platysma. Injection that is too superficial can cause dermal necrosis or ulceration. Injection that is too deep can injure muscle, gland, vessel, or nerve. Resistance during needle advancement may indicate fascial or non-fat tissue. Palpation and anatomical knowledge remain essential, and ultrasound may be valuable when tissue planes are uncertain, although routine ultrasound-guided DCA injection has not yet been validated in large controlled studies.

The marginal mandibular nerve is particularly important. Injury can produce an asymmetric smile or weakness of lower-lip depressors. The prescribing information defines an avoidance zone below the mandibular border and recommends restricting treatment to the target submental area. In the jowl, the nerve, facial vessels, retaining structures, and thin fat compartments make extrapolation from central submental treatment hazardous. A product that is relatively confined in thick central fat may contact vulnerable structures in a thin lateral compartment.

### 7.3. Expected Reactions and Serious Complications

Common reactions include edema, pain, hematoma or bruising, numbness, erythema, and induration. These typically diminish over time and are linked to injection trauma and intended inflammation. Patient-experience protocols involving cold, local anesthesia, and clear counseling can improve tolerability [50]. Management should not suppress the tissue response indiscriminately without evidence, but symptom control is appropriate when it does not compromise safety assessment.

Less common but clinically important events include marginal mandibular nerve paresis, dysphagia, vascular injury, alopecia, ulceration, necrosis, infection, and prolonged nodularity. Technique, superficial placement, excessive overlap, treatment outside the intended anatomical zone, and failure to recognize non-fat structures can contribute. Published management guidance emphasizes early recognition and complication-specific care rather than assuming all post-treatment swelling is benign [51,52].

Alopecia is biologically plausible when inflammation, vascular compromise, or direct drug exposure affects hair follicles in the treatment field. Ulceration and necrosis suggest intradermal or very superficial exposure, vascular injury, or excessive local concentration. Dysphagia may occur in association with edema or injury near swallowing-related structures. Infection can follow barrier disruption or necrosis. Because postmarketing reports lack a denominator, they cannot establish incidence, but they expand the range of plausible complications beyond those captured in trials.

### 7.4. Off-Label Regional Use

DCA has been used outside the submental region, including the jowl, upper arm, abdomen, flank, thigh, knee, and other localized deposits. Real-world reports suggest potential efficacy, but evidence quality is lower and protocols are heterogeneous [53]. The United States prescribing information states that safe and effective use outside the submental region has not been established and is not recommended [29].

Regional anatomy changes the risk–benefit calculation. Body compartments may contain larger fat volumes and require higher total dose. Thin facial compartments may place nerves and vessels close to the depot. Lymphatic drainage, skin thickness, fibrous septa, mobility, and healing differ by region. A dose per unit area validated under the chin cannot be assumed to produce the same distribution or systemic exposure in the abdomen or upper arm. Off-label studies should report concentration, volume per point, spacing, total dose, ultrasound-measured fat thickness, needle depth, session interval, and adverse-event surveillance in enough detail to permit mechanistic interpretation.

Table 4 summarizes the evidence hierarchy and its translational meaning. The strongest efficacy evidence concerns labeled submental use. Mechanistic certainty is strongest for membrane injury and histological remodeling. Evidence is weakest for individualized metabolic prediction and broad body-area dosing.

## 8. A Metabolomics and Lipidomics Framework for Injectable DCA

### 8.1. Why Omics Are Needed

The rationale for applying metabolomics and lipidomics to injectable DCA arises from properties that are specific to this intervention. DCA is both an endogenous secondary bile acid and an administered cytolytic drug. Consequently, treatment-related systemic exposure must be distinguished from a pre-existing bile-acid pool that varies with fasting state, diet, microbial activity, hepatic handling, and enterohepatic cycling. After injection, the administered molecule enters this endogenous pool and may be reconjugated to glycodeoxycholic acid and taurodeoxycholic acid. Measurement of total DCA alone may therefore be insufficient to distinguish baseline physiology, acute drug exposure, and subsequent hepatic processing.

The local response creates a second DCA-specific analytical challenge. Concentrated DCA disrupts cell membranes and releases intracellular lipids, membrane components, ions, and damage-associated molecular patterns. Conventional clinical endpoints—including rating scales, ultrasound thickness, magnetic resonance imaging, three-dimensional photography, and satisfaction—describe anatomical outcome but do not establish how local exposure produces tissue clearance. Similarly, conventional plasma lipid measurements may fail to detect short-lived or locally confined changes in non-esterified fatty acids, glycerol, oxylipins, lysophospholipids, acylcarnitines, or bile-acid conjugates.

A multiscale framework is therefore relevant to DCA because it can connect local drug exposure with membrane injury, lipid release, inflammatory resolution, fibrovascular remodeling, systemic bile-acid handling, and final contour outcome. At the local level, measurements should address membrane damage, extracellular lipid, inflammatory signaling, and matrix turnover. At the systemic level, assays should distinguish unconjugated DCA from conjugated species and baseline variation. Imaging should quantify edema, perfusion, tissue stiffness, and adipose thickness. Integration of these domains may identify early signatures associated with effective clearance, prolonged inflammation, or complications.

Metabolomics studies must nevertheless use standardized terminology and transparent reporting. The Metabolomics Standards Initiative proposed minimum reporting requirements for chemical analysis and data interpretation, while established LC–MS protocols emphasize extraction controls, pooled quality-control samples, blanks, internal standards, randomized run order, and assessment of instrument drift [54,55,56]. These safeguards are especially important in the acute DCA setting because the largest exposure-related changes may occur within minutes.

### 8.2. Biospecimen Strategy and Sampling Windows

A minimum plasma protocol should include more than a single pre- and post-treatment sample. Because peak systemic DCA occurs rapidly, samples at baseline, 15–30 min, 60 min, 2 h, 6 h, and 24 h are appropriate for pharmacokinetic characterization. Samples at days 3, 7, 28, and 84 can assess inflammatory resolution and remodeling. To characterize endogenous variability, at least two baseline samples under standardized fasting conditions—or a pre-treatment time series on a separate day—would be preferable.

Local sampling may provide greater mechanistic specificity than plasma analysis, but the available methods are not interchangeable. Microdialysis is best suited to serial measurement of recoverable water-soluble interstitial analytes during the first hours, including glycerol, glucose, lactate, and selected small inflammatory mediators. Its recovery of hydrophobic lipids, protein-bound bile acids, and large proteins is limited and depends on membrane characteristics, perfusate composition, flow rate, and probe recovery. Probe insertion also causes local injury and therefore requires appropriate baseline and procedural controls.

Suction-blister fluid provides a less invasive source of superficial interstitial proteins and inflammatory mediators but may not represent the deeper subcutaneous injection depot. Fine-needle aspiration can retrieve extracellular lipid material and cellular components for cytology, immune-cell characterization, and selected molecular assays, although recovery is spatially limited and may vary with tissue consistency. Timed core biopsy or planned tissue excision provides the most informative material for histology, immunohistochemistry, cellular phenotyping, extracellular-matrix assessment, and spatial omics, but carries greater invasiveness, sampling bias, scarring, and ethical complexity. Studies involving patients already undergoing abdominoplasty or neck surgery may permit planned injection and excision, as demonstrated in prior histological work.

The sampling method should therefore be selected according to the biological question. Microdialysis is most appropriate for early soluble interstitial changes; blister fluid for superficial inflammatory signaling; aspiration for extracellular lipid and cellular content; and tissue biopsy for spatially resolved injury, immune infiltration, and matrix remodeling. Results obtained from one compartment should not be assumed to represent another.

Urine and stool have secondary roles. Urine may reflect systemic inflammatory or metabolic responses but is unlikely to quantify local adipose clearance directly. Stool bile-acid profiling could reveal baseline microbial DCA production and host–microbial phenotype; however, the administered drug is expected to merge with the enterohepatic pool, and fecal measurements cannot readily separate exogenous from endogenous DCA without isotope labeling. Stable-isotope-labeled DCA, if safe and ethically justified, would be the most direct method to track drug fate independently of the background pool.

Standardization is essential. Participants should have controlled fasting duration, meal timing, alcohol intake, exercise, and medication use. Antibiotics, probiotics, bile-acid sequestrants, metformin, glucagon-like peptide-1 receptor agonists, and bariatric surgery can influence bile-acid or metabolic profiles. Menstrual phase, circadian timing, and recent weight change should be recorded. Samples must be processed rapidly at controlled temperature, because lipases and oxidation can alter lipid mediators ex vivo.

### 8.3. Targeted Bile-Acid Profiling

A targeted bile-acid panel should quantify unconjugated DCA, glycodeoxycholic acid, taurodeoxycholic acid, cholic acid, chenodeoxycholic acid, lithocholic acid, ursodeoxycholic acid, and their major conjugates. Reporting only total bile acids would obscure drug-specific kinetics. Calibration should account for matrix effects and isomeric separation. Because some bile-acid species share mass transitions, chromatographic resolution and authentic standards are required.

Useful derived variables include the DCA fraction of the total bile-acid pool, DCA-to-cholic-acid ratio, conjugated-to-unconjugated DCA ratio, and area under the concentration–time curve. These measures may distinguish baseline microbial production, acute drug exposure, and hepatic reconjugation. A paired stool profile could estimate the participant’s intrinsic DCA-producing phenotype. Measurement of FGF19 and 7α-hydroxy-4-cholesten-3-one may provide indirect information about enterohepatic feedback and bile-acid synthesis, although the small injected dose may not produce a detectable response.

Interpretation should avoid a common error: a post-treatment rise in plasma DCA does not automatically imply systemic toxicity. Toxicity depends on free concentration, duration, protein binding, organ exposure, and host susceptibility. Conversely, return of total DCA to the endogenous range does not prove absence of downstream effects. Conjugated species, local metabolites, and transient signaling may persist after the parent concentration has normalized.

### 8.4. Lipidomics of Adipocyte Disruption and Clearance

Adipocyte destruction should create a characteristic lipid trajectory. Early local samples may contain glycerol, non-esterified fatty acids, monoacylglycerols, diacylglycerols, triacylglycerols, cholesterol, cholesteryl esters, phosphatidylcholines, phosphatidylethanolamines, sphingomyelins, ceramides, and lysophospholipids. Membrane fragmentation may increase lysophosphatidylcholine and lysophosphatidylethanolamine through phospholipase activity. Oxidative stress and immune activation may generate eicosanoids and other oxylipins. Macrophage uptake and mitochondrial processing may alter acylcarnitines.

Temporal direction is likely more informative than a single concentration. A rapid local rise in free fatty acids and membrane lipids may mark cytolysis. Subsequent elevation of oxidized lipids and inflammatory mediators may reflect innate immune processing. Decline of extracellular lipid alongside changes in macrophage-associated metabolites may indicate clearance. Persistent ceramide, oxidized phospholipid, or pro-inflammatory oxylipin signals could identify delayed resolution or excessive injury.

Plasma changes will be smaller than local changes and may require highly sensitive targeted assays. Lipoprotein fractionation could determine whether released lipid is transported in very-low-density lipoprotein, low-density lipoprotein, or high-density lipoprotein fractions. Apolipoprotein B, apolipoprotein A-I, particle number, and remnant cholesterol may be more informative than total cholesterol. Non-esterified fatty acids should be measured with precise timing because they are influenced by fasting, stress, catecholamines, and insulin.

A key analytical problem is distinguishing lipid released from treated adipocytes from ordinary systemic flux. Stable-isotope labeling of dietary fatty acids is impractical for routine studies, but local microdialysis and paired systemic sampling can help. Another approach is to compare the fatty-acid composition of baseline adipose biopsy with post-treatment plasma or local fluid. Adipose depots have characteristic fatty-acid patterns, although overlap with dietary and hepatic sources remains substantial.

### 8.5. Inflammatory and Matrix-Remodeling Biomarkers

Metabolomics alone will not capture the full response. A complementary protein panel should include interleukin-6, tumor necrosis factor, monocyte chemoattractant protein-1/CCL2, C-reactive protein, select chemokines, adiponectin, leptin, and markers of endothelial activation. Local concentrations are expected to be more informative than plasma. Lactate dehydrogenase, cell-free DNA, high-mobility group box 1, and potassium may reflect early membrane injury, although specificity is limited.

Matrix remodeling can be studied through procollagen peptides, collagen degradation products, matrix metalloproteinases, tissue inhibitors of metalloproteinases, hyaluronan fragments, and elastin-derived peptides. Ultrasound elastography can provide a non-invasive correlate of tissue stiffness. Contrast-enhanced ultrasound or dynamic imaging may assess perfusion and neovascularization. The goal is not simply to prove that fibrosis occurs, but to determine whether the magnitude and organization of matrix change predict desirable contour stabilization or undesirable induration.

Single-cell RNA sequencing, spatial transcriptomics, and imaging mass spectrometry could identify macrophage, fibroblast, endothelial, and progenitor-cell states around the injection core. These methods are expensive and require tissue, but a small mechanistic study could be transformative. Spatial data would test whether DCA creates a central cytolytic zone surrounded by metabolically active repair zones and whether repeat treatment changes that architecture.

### 8.6. Analytical Quality Control and Data Integration

Untargeted LC–MS can discover unexpected metabolites, but it should be paired with targeted quantification of DCA and predefined lipid classes. Quality-control samples should be injected at regular intervals, and batch effects should be modeled rather than ignored. System suitability, pooled biological QC, internal standards spanning chemical classes, extraction blanks, and duplicate samples are essential. Guidance for clinical metabolomics emphasizes that analytical precision and reproducibility must be demonstrated before biological interpretation [57,58].

Data analysis should prioritize longitudinal within-person changes. Mixed-effects models can account for repeated measures and variable numbers of treatment sessions. Multiple-testing correction is required for untargeted features. Pathway enrichment should not be treated as proof of mechanism when metabolite identification is uncertain. Features should be assigned confidence levels, and key findings should be validated with authentic standards.

Integration with imaging and clinical outcomes can use multiblock methods or carefully regularized machine learning, but sample size must match model complexity. A practical primary endpoint could be percentage reduction in ultrasound-measured fat thickness at 12 weeks. Candidate predictors could include early local DCA exposure, a lipid-release score, an inflammatory-resolution score, and baseline fibrotic phenotype. Models should be externally validated before use for dose selection (Figure 4).

Table 5 presents a modular protocol linking each biospecimen method to the analytical readouts and biological questions for which it is best suited. A basic clinical study may combine standardized plasma bile-acid profiling with imaging and clinical outcomes, whereas a mechanistic study may add microdialysis, aspiration, biopsy, or planned tissue excision according to the required sampling depth, cellular content, and analyte class.

## 9. Critical Knowledge Gaps and Research Priorities

Section 8 outlines the biospecimens, analytical methods, and quality-control procedures that could be used to study injectable DCA. The present section does not repeat that methodological framework; instead, it identifies the DCA-specific questions that those methods should address. These priorities concern the local therapeutic window, the distinction between adaptive and pathological remodeling, interindividual heterogeneity, clinically meaningful outcome measurement, and the validation required before biomarker-guided dosing can be considered.

### 9.1. Defining the Effective and Harmful Exposure Thresholds

The field lacks a quantitative local pharmacology model linking injected DCA concentration and depot geometry to the exposure experienced by adipocyte, dermal, neural, vascular, and muscular membranes. The sampling and imaging approaches outlined in Section 8 could determine whether efficacy and toxicity are associated primarily with peak local concentration, duration of exposure, depot overlap, or total treated volume. Establishing these relationships is necessary before dosing can be rationally adapted across anatomical compartments. A related priority is to define a therapeutic window for different anatomical compartments. The submental fat layer is not a universal model. Thin jowl fat, fibrous knee fat, mobile upper-arm tissue, and broad abdominal depots have different boundaries and diffusion pathways. Ultrasound-measured thickness and septal pattern should be incorporated into protocol design. Regional studies should therefore begin with conservative dosing, ultrasound-based anatomical characterization, and preplanned stopping rules rather than applying the submental grid without validation.

### 9.2. Distinguishing Desirable Remodeling from Pathological Fibrosis

Septal thickening may contribute to contour stabilization, whereas persistent induration and nodularity may indicate excessive or disorganized remodeling. The imaging, matrix-marker, and tissue methods described in Section 8.5 and Section 8.6 could help distinguish these outcomes, but no validated threshold currently separates adaptive repair from clinically problematic fibrosis. Repeat treatment is particularly relevant because a subsequent injection enters tissue with fewer adipocytes and altered extracellular matrix, vascularity, compliance, and diffusion. Studies should compare untreated and previously treated tissue rather than assume that successive sessions are biologically equivalent.

### 9.3. Understanding Immune and Metabolic Heterogeneity

The macrophage response after DCA injection has been observed histologically but has not been fully phenotyped. The cellular and spatial approaches described in Section 8.5 could determine whether delayed swelling or nodularity corresponds to persistent inflammatory or reparative cell states. Patient-level factors—including insulin resistance, liver steatosis, dyslipidemia, medications, and microbiome composition—may also influence bile-acid kinetics and tissue repair. These possibilities remain hypotheses and require prospective DCA-specific investigation rather than extrapolation from BMI or general obesity studies.

### 9.4. Improving Clinical Outcome Measurement

Clinical rating scales and photography should be complemented by objective measures of fat thickness, tissue stiffness, and surface contour. As outlined in Section 8, ultrasound, magnetic resonance imaging, and three-dimensional photography provide different but complementary outcome domains. Adverse-event reporting should also distinguish events according to onset, duration, severity, functional impact, and anatomical localization. Standardized outcomes and region-specific denominators are particularly important for off-label treatment.

### 9.5. Toward Biomarker-Guided Dosing

The long-term objective is to combine anatomical assessment with validated biological measurements rather than replace clinical judgment. Ultrasound thickness, local exposure, lipid-release markers, and inflammatory-resolution profiles are candidate variables, but no metabolomic biomarker is currently validated to predict DCA efficacy or safety. The framework in Section 8 should therefore be regarded as a discovery strategy. Candidate biomarkers must first demonstrate analytical validity, temporal reproducibility, association with objective outcomes, and independent external validation before they can guide dose, spacing, or retreatment decisions.

## 10. Conclusions

DCA is a model of how a single metabolite can occupy distinct biological regimes. Within the enterohepatic system, it is a microbial–host co-metabolite whose effects are shaped by conjugation, transport, protein binding, receptor signaling, and feedback regulation. Within a concentrated subcutaneous depot, it is primarily a membrane-active cytolytic agent. The therapeutic endpoint emerges not from selective metabolic stimulation of viable adipocytes, but from controlled adipocyte destruction followed by immune clearance and stromal repair.

This framework resolves several ambiguities. “Adipocytolysis” is more accurate than “lipolysis” for the initiating mechanism. Apparent adipose selectivity is relative and depends on tissue architecture and protein buffering. Fibrovascular remodeling may contribute to contour stability, but it should not automatically be equated with dermal rejuvenation. Pharmacokinetic reassurance from labeled submental dosing should not be extrapolated without evidence to large-area or high-dose body treatment.

The next phase of research should connect local exposure to systemic handling and long-term tissue outcome. Targeted bile-acid profiling, lipidomics, inflammatory markers, spatial tissue analysis, ultrasound, and validated clinical measures can provide that connection. A rigorous multiscale approach would allow injectable DCA to be studied not merely as an aesthetic procedure, but as a controlled metabolic and tissue-remodeling perturbation. That perspective offers the best route toward individualized dosing, improved safety, and a more precise understanding of the interface between bile-acid biology and adipose-tissue remodeling.

## Figures and Tables

**Figure 1:**
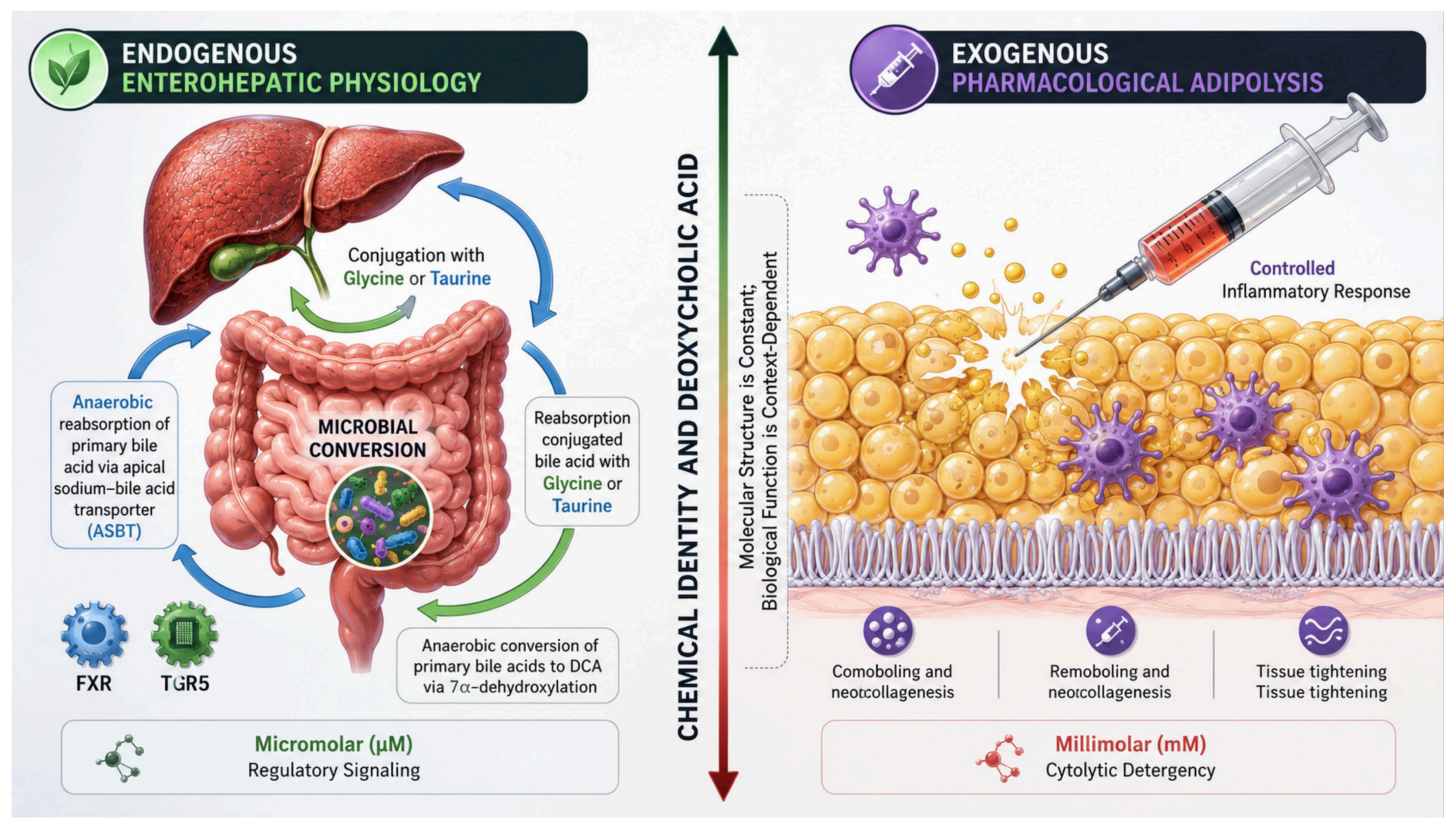
Conceptual overview of the dual biological context of deoxycholic acid. Endogenous DCA is formed through microbial conversion of host-derived cholic acid, reabsorbed into the portal circulation, reconjugated in the liver, and returned to the enterohepatic bile-acid pool, where its biological actions are shaped by concentration, transport, protein binding, and receptor signaling. Concentrated injectable DCA instead produces local membrane disruption and adipocytolysis, followed by lipid release, inflammatory-cell recruitment, macrophage-mediated clearance, fibrovascular remodeling, and contour reduction. The figure provides an integrative conceptual comparison rather than a complete chemical reaction scheme. DCA, deoxycholic acid; FXR, farnesoid X receptor; TGR5/GPBAR1, G-protein-coupled bile-acid receptor 1.

**Figure 2:**
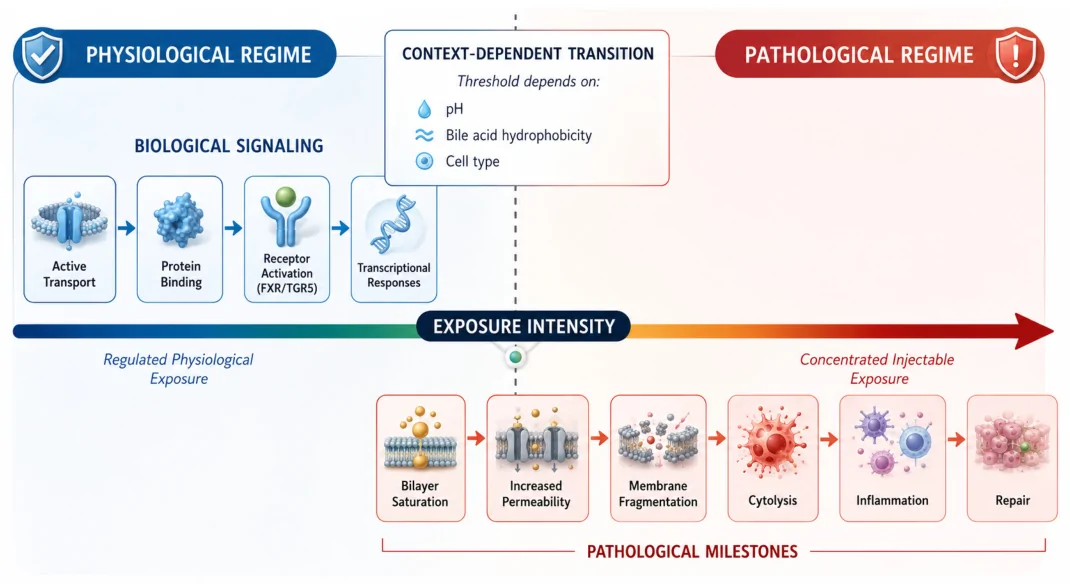
Exposure-dependent continuum from physiological bile-acid signaling to concentrated membrane injury. Under regulated physiological conditions, DCA activity is influenced by transport, protein binding, receptor availability, and cellular context. FXR functions as a nuclear bile-acid receptor, whereas TGR5/GPBAR1 mediates membrane-receptor signaling through cyclic adenosine monophosphate-dependent pathways. Under concentrated injectable exposure, bile-salt partitioning into phospholipid bilayers can progress through bilayer saturation, increased permeability, membrane fragmentation, cytolysis, inflammation, and repair. The transition is context-dependent and should not be interpreted as a universal concentration threshold. DCA, deoxycholic acid; FXR, farnesoid X receptor; TGR5/GPBAR1, G-protein-coupled bile-acid receptor 1.

**Figure 3:**
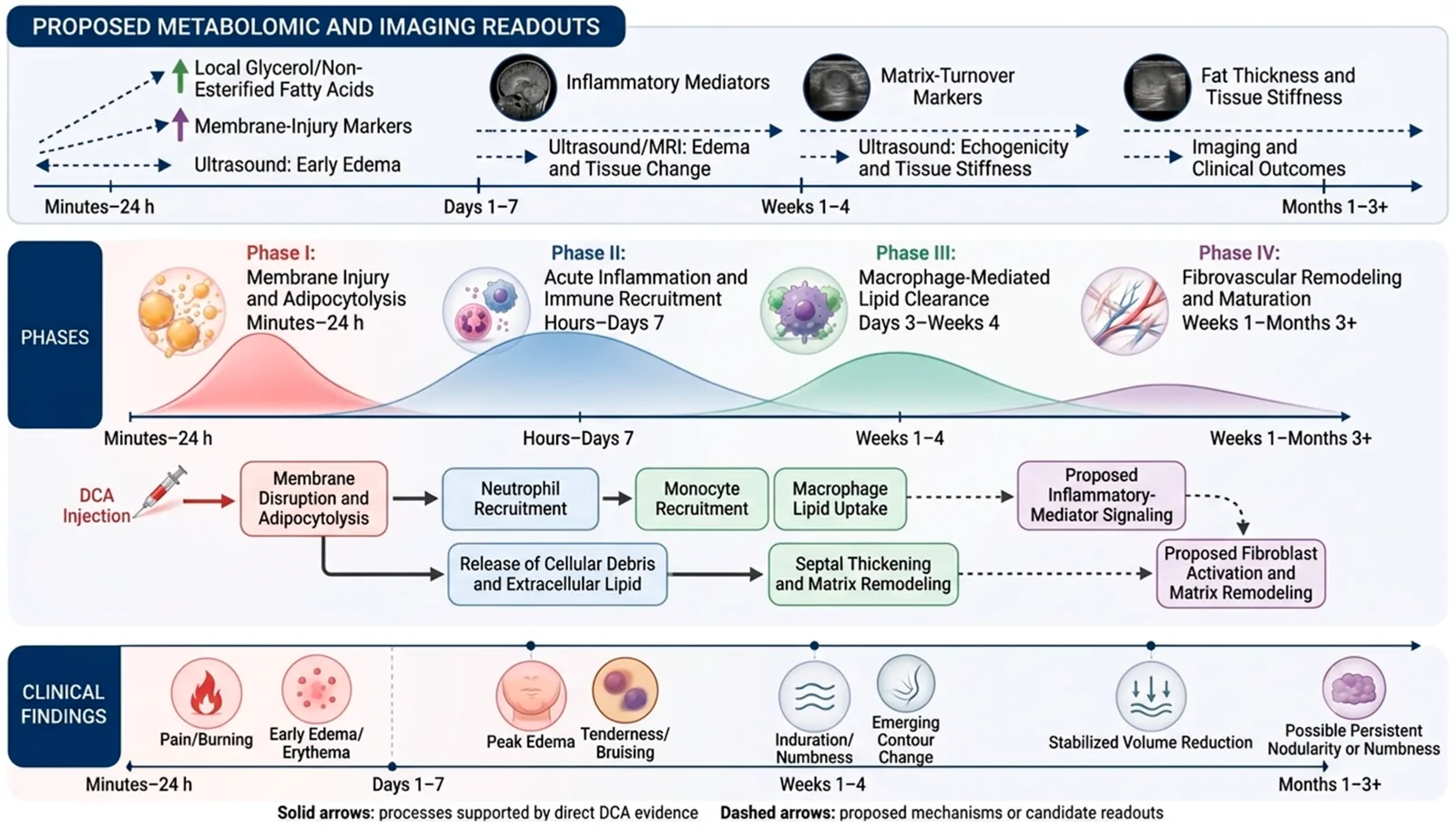
Temporal sequence of local tissue responses, proposed analytical readouts, and clinical findings following injectable deoxycholic acid administration. Membrane disruption and adipocytolysis occur during the initial minutes to 24 h and are followed by acute inflammation and immune-cell recruitment over the subsequent hours and days. Monocyte recruitment and macrophage-mediated lipid uptake contribute to clearance during the following days and weeks, while septal thickening and fibrovascular remodeling develop over weeks to months. The (**upper panel**) presents proposed metabolomic and imaging readouts aligned with these overlapping phases, whereas the (**lower panel**) summarizes their expected clinical correlates. Solid arrows indicate processes supported by direct DCA evidence, and dashed arrows indicate proposed mechanisms or candidate analytical readouts. The indicated intervals represent overlapping biological phases rather than rigid temporal boundaries. DCA, deoxycholic acid; MRI, magnetic resonance imaging.

**Figure 4:**
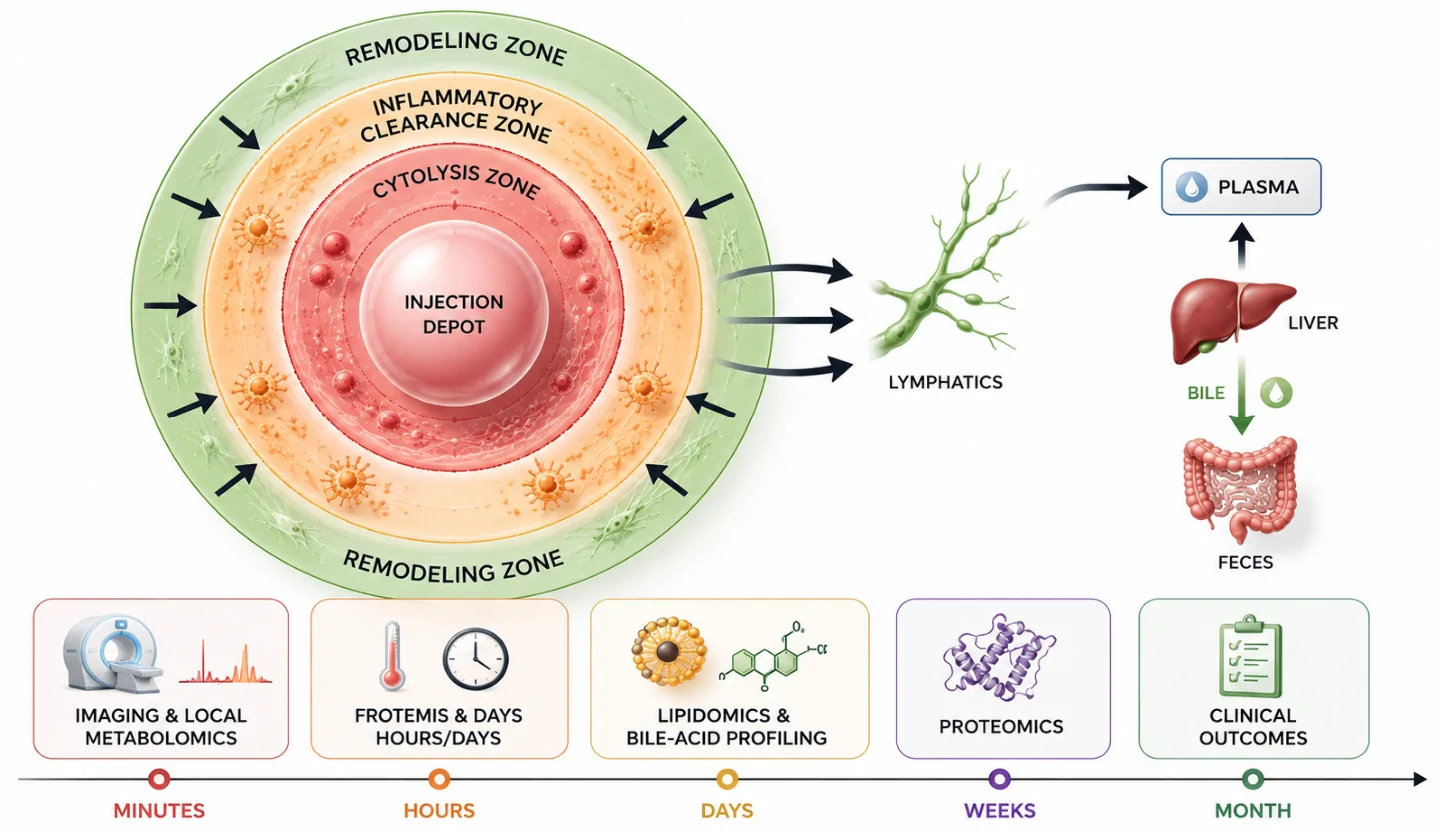
Proposed local-to-systemic framework for studying injectable DCA. A central injection depot is surrounded conceptually by zones of cytolysis, inflammatory clearance, and remodeling. Local fluid and tissue measurements are linked to lymphatic transport, plasma exposure, hepatic handling, bile, and fecal elimination. The sampling timeline extends from minutes and hours for acute pharmacokinetics and injury markers to days and weeks for inflammatory clearance and months for imaging and clinical outcomes. Analytical modules include targeted bile-acid profiling, lipidomics, inflammatory and matrix biomarkers, tissue phenotyping, imaging, and clinical assessment. The framework is hypothesis-generating and does not imply that each proposed pathway or biomarker has been validated clinically. DCA, deoxycholic acid.

**Table 1:** Comparative biological contexts of endogenous and injectable deoxycholic acid.

Domain	Endogenous DCA	Injectable DCA	Translational Implication
Origin	Microbial 7α-dehydroxylation of host-derived cholic acid	Synthetic DCA in a standardized pharmaceutical formulation	Chemical identity is shared, but exposure context is not
Primary compartment	Intestinal lumen, portal blood, liver, bile, enterohepatic pool	Restricted extracellular depot in subcutaneous fat	Local concentration and diffusion dominate the initial response
Typical biological role	Mixed-micelle formation, bile-acid pool composition, receptor signaling	Physical membrane injury and adipocytolysis	The therapeutic mechanism is cytolytic rather than classical lipolysis
Major moderators	Microbiome, diet, transit, conjugation, transporters, hepatic feedback	Concentration, volume, dose per area, injection depth, tissue thickness, protein binding	Technique and anatomy are mechanistic variables
Time scale	Continuous cycling with meal- and circadian-related variation	Acute exposure followed by days-to-weeks of inflammation and remodeling	Sampling must cover minutes through months
Systemic interpretation	Marker and mediator of microbial–host metabolism	Transient addition to an endogenous, highly variable pool	Baseline profiling and isotope-aware methods are desirable

**Table 2:** Proposed temporal sequence after local DCA injection.

Approximate Phase	Dominant Tissue Events	Clinical Correlates	Candidate Measurements
Minutes–24 h	Depot diffusion, membrane destabilization, adipocyte injury, vascular permeability, release of intracellular metabolites and DAMPs	Burning, pain, edema, erythema, early bruising	Plasma/local DCA, GDCA/TDCA, glycerol, non-esterified fatty acids, LDH, potassium, ultrasound edema
Days 1–7	Neutrophil and monocyte recruitment, macrophage lipid uptake, extracellular lipid pools, evolving hemorrhage and repair	Peak swelling, tenderness, firmness, numbness	Cytokines, chemokines, oxylipins, lysophospholipids, macrophage markers, contrast-enhanced ultrasound
Weeks 1–4	Resolution of acute inflammation, fibroblast activation, septal thickening, neovascularization, fat-lobule atrophy	Decreasing edema; emerging contour change; residual induration	Collagen-turnover markers, elastography, vascular imaging, targeted lipid mediator panel
Months 1–3+	Matrix maturation and stabilization of volume reduction	Final contour assessment; possible persistent numbness, nodularity, or alopecia in complicated cases	3D photography, MRI or ultrasound fat thickness, tissue stiffness, patient-reported outcomes

**Table 3:** Clinical evidence and quantitative treatment characteristics of injectable DCA for submental fat reduction.

Evidence Source	Design/Context	Quantitative Treatment or Follow-Up Information Available in This Review	Main Finding	Principal Limitation
European phase III studies [40,41,42]	Randomized, placebo-controlled phase III studies of submental fullness	Controlled dose-ranging and phase III treatment; study-specific numerical response rates are not reproduced in the present narrative synthesis	Clinician- and patient-rated submental fat reduction was greater with ATX-101 than with placebo	Selected populations; local treatment reactions; restricted to submental use
REFINE trials [43,44]	Multicenter randomized, double-blind, placebo-controlled phase III studies	DCA 2 mg/cm^2^; outcomes included validated rating scales and MRI-based fat-volume assessment	Confirmed improvement in moderate-to-severe submental fullness compared with placebo	Multiple treatment sessions and expected local reactions
Pooled REFINE analysis [45]	Pooled phase III analysis by treatment session	Progressive response across treatment sessions	Supports individualized cessation after the desired response	Pooled analysis; does not establish efficacy outside the studied regimen
Long-term follow-up [46]	Follow-up of phase III responders	Follow-up for up to 3 years	Improvement persisted in many responders	Follow-up participation and responder selection may influence estimates
Open-label study [47]	Prospective open-label submental treatment	Follow-up reported at 12 months	Supported continued clinical benefit after treatment	No placebo comparator
Consecutive real-world series [48,49]	Real-world neck-contouring experience	100 consecutive patients; updated follow-up extending to 2 years	Supported feasibility and durability in experienced practice	Uncontrolled design and potential selection and assessment bias
Regulatory dosing information [29]	Approved submental-use instructions	10 mg/mL; 0.2 mL per injection; approximately 1 cm spacing; 2 mg/cm^2^; up to 50 injections; maximum 100 mg per session; at least 1 month between sessions	Defines the standardized exposure used to support labeled clinical translation	Does not establish safety or efficacy for non-submental or large-area treatment

**Table 4:** Translational evidence for injectable DCA.

Evidence Domain	Representative Evidence	What Is Supported	Principal Limitation
Membrane mechanism	Cell, ex vivo, and animal studies of deoxycholate and related formulations [4,5,8]	Concentrated deoxycholate disrupts membranes and is not intrinsically adipocyte-receptor selective	Formulation and exposure conditions differ among studies
Human tissue response	Timed histology after ATX-101 [6]	Acute adipocytolysis followed by macrophage infiltration, septal thickening, neovascularization, and lobular atrophy	Small study; abdominal tissue; limited molecular phenotyping
Pharmacokinetics	Phase I plasma DCA and metabolic markers [33,39]	Rapid transient systemic exposure and no major conventional metabolic signal at labeled-scale dosing	Small cohorts; limited analytes; not designed for large-area treatment
Clinical efficacy	European phase III and REFINE trials [40,41,43,44]	Improvement in moderate-to-severe submental fullness versus placebo	Selected patients; treatment-specific downtime; submental indication only
Durability	Long-term follow-up [46]	Benefit persists in many responders for up to three years	Follow-up participation and responder selection may influence estimates
Off-label use	Case series and real-world reports [53]	Feasibility in selected non-submental regions	Heterogeneous protocols, limited controls, uncertain dose scaling
Serious adverse events	Label, case reports, and systematic safety review [29,52]	Nerve injury, dysphagia, alopecia, ulceration/necrosis, vascular injury, and infection are recognized risks	True incidence and region-specific denominators are uncertain

**Table 5:** Proposed multiscale protocol for studying injectable DCA.

Module/Method	Samples and Timing	Appropriate Analytes/Readouts	Principal Biological Question	Important Limitation
Baseline phenotype	Two fasting plasma samples; stool; ultrasound; metabolic laboratory tests	Individual bile-acid species, glucose/insulin, lipids, liver tests, fecal bile acids, fat thickness and tissue stiffness	Does baseline microbial–metabolic phenotype predict DCA exposure or response?	Endogenous bile-acid variability requires standardized fasting and collection
Acute systemic pharmacokinetics	Plasma at baseline, 15–30 min, 1 h, 2 h, 6 h, and 24 h	DCA, GDCA, TDCA, total bile-acid profile, and free fraction where feasible	How rapidly does administered DCA enter and leave systemic circulation?	Administered DCA merges with a variable endogenous pool
Microdialysis	Local interstitial sampling during the first hours	Glycerol, glucose, lactate, selected small inflammatory mediators, and validated recoverable analytes	What early soluble interstitial changes accompany local cytolysis?	Limited and probe-dependent recovery of hydrophobic lipids, protein-bound bile acids, and large proteins; insertion-related injury
Suction-blister fluid	Superficial fluid sampling during early inflammation	Superficial inflammatory proteins, selected cytokines and chemokines, LDH, and soluble injury markers	Can superficial interstitial signaling be monitored less invasively?	May not represent the deeper subcutaneous injection depot
Fine-needle aspiration	Targeted local sampling during the first days	Extracellular lipid material, immune cells, cytology, and selected cellular markers	Which cellular and lipid components accumulate at the treated site?	Spatially limited and variable material recovery
Core biopsy or planned excision	Tissue obtained at predefined acute and remodeling time points	Histology, immunohistochemistry, macrophage and stromal phenotypes, extracellular matrix, spatial transcriptomics, and imaging mass spectrometry	How are injury, immune clearance, and fibrovascular remodeling spatially organized?	Invasive; potential scarring, sampling bias, and ethical constraints
Inflammatory clearance	Local and plasma sampling at days 3 and 7	Oxylipins, lysophospholipids, cytokines, chemokines, and macrophage-associated markers	Which response is associated with efficient clearance rather than prolonged inflammation?	Candidate biomarkers are not clinically validated
Remodeling	Imaging and optional tissue at days 28 and 84	Fat thickness, elastography, perfusion, collagen-turnover markers, and spatial omics	Does organized fibrovascular remodeling predict durable contour reduction?	Tissue access may be limited; matrix biomarkers are not DCA-specific
Clinical outcome	3D photography, validated scales, and patient-reported outcomes at 12 weeks after final treatment	Objective contour change, adverse events, downtime, and satisfaction	Can early molecular signatures improve patient selection and dosing?	Predictive models require independent validation

## Data Availability

No new data were created or analyzed in this study. Data sharing is not applicable to this article.

## Abbreviations

Abbreviations used in this review.

ATX-101	Standardized injectable formulation of synthetic deoxycholic acid
DAMP	Damage-associated molecular pattern
DCA	Deoxycholic acid
ECM	Extracellular matrix
FGF19	Fibroblast growth factor 19
FXR	Farnesoid X receptor
GDCA	Glycodeoxycholic acid
LC–MS	Liquid chromatography–mass spectrometry
SMF	Submental fat
TDCA	Taurodeoxycholic acid
TGR5/GPBAR1	G-protein-coupled bile-acid receptor 1
UPLC–MS	Ultra-performance liquid chromatography–mass spectrometry
